# Internal plate fixation versus plaster in displaced complete articular distal radius fractures, a randomised controlled trial

**DOI:** 10.1186/s12891-016-0925-y

**Published:** 2016-02-09

**Authors:** Marjolein A. M. Mulders, Monique M. J. Walenkamp, J. Carel Goslings, Niels W. L. Schep

**Affiliations:** Trauma Unit, Department of Surgery, Academic Medical Center, University of Amsterdam, P.O. Box 22660, 1100 DD Amsterdam, The Netherlands; Department of Surgery, Maasstad Hospital, P.O. Box 9100, 3007 AC Rotterdam, The Netherlands

**Keywords:** Distal radius fracture, Articular, Displaced, Plaster, Open reduction internal fixation, Wrist function, PRWE, Randomised controlled trial

## Abstract

**Background:**

Of all distal radius fractures, 25 % are complete articular fractures (AO/OTA type C fractures). Two thirds of those fractures are displaced and require reduction. According to several International Guidelines, adequately reduced intra-articular distal radius fractures are best treated non-operatively with plaster immobilisation, while surgical fixation is suggested only when the articular step exceeds 2 mm after reduction. However, these recommendations are based on studies that did not differentiate between intra- and extra-articular distal radius fractures. Thus, no clear consensus about the best treatment for patients with displaced intra-articular distal radius fractures can be reached. Despite the lack of evidence, an increase in internal fixation of intra-articular distal radius fractures has been observed over the last decade. The aim of this study is to determine the difference in functional outcome following open reduction and plate fixation compared with non-operative treatment with closed reduction and plaster immobilisation in patients with a displaced intra articular distal radius fracture.

**Methods/Design:**

This multicentre randomised controlled trial will randomise between open reduction and internal plate fixation (intervention group) and closed reduction and plaster immobilisation (control group). All consecutive adult patients from 18 to 65 years with a displaced intra-articular distal radius fracture (AO/OTA type C), which has been adequately reduced at the Emergency Department according to the Dutch National Guidelines, are eligible for inclusion in this study. The primary outcome is function and pain of the wrist assessed with the Patient-Rated Wrist Evaluation score (PRWE). Secondary outcomes are the Disability of the Arm, Shoulder and Hand score (DASH), pain, quality of life (SF-36), range of motion, grip strength, radiological parameters, complications, crossovers and cost-effectiveness of both treatments. A total of 90 patients will be included in this study.

**Discussion:**

Although displaced intra-articular distal radius fractures are common, there is still no evidence on the optimal treatment for these fractures in patients aged 18 to 65 years. Therefore we aim to determine the difference in functional outcome between open reduction and plate fixation and closed reduction and plaster immobilisation.

**Trial registration:**

This study is registered at ClinicalTrials.gov (NCT02651779) on January 4^th^ 2016.

## Background

Distal radius fractures are the most common fractures in the adult population [1]. Recently, Bentohami et al. and Mellstrand-Navarro et al. found an overall incidence of distal radius fractures of respectively 24 and 32 per 10.000 persons each year [2, 3]. Two thirds of these fractures are displaced and require reduction [4]. Of all distal radius fractures, 25 % are complete articular fractures (AO/OTA type C fractures) [2, 5].

According to the Dutch National Guidelines, displaced intra-articular distal radius fractures, following adequate reduction confirmed on X-ray, are best treated non-operatively with plaster immobilisation [6]. Moreover, the American Academy of Orthopaedic Surgeons (AAOS) Clinical Practice Guideline only suggests surgical treatment when the articular step, after reduction, exceeds 2 mm [7]. However, these recommendations are based on studies that did not differentiate between intra- and extra-articular distal radius fractures. Therefore, no clear consensus about the best treatment for displaced distal radius fractures exists. For the displaced extra-articular fractures (AO/OTA type A2 and A3) we already initiated the VIPER trial [8]. However, there is still no evidence on the optimal treatment of the displaced intra-articular fractures.

Despite the lack of evidence, an increase of open reduction and internal fixation (ORIF) of intra-articular distal radius fractures has been observed over the last decade [9–11]. Both Koval et al. and Matilla et al. described doubling of ORIF over a 10 year period, respectively from 23.9 surgical operations per 100.000 persons each year to 47.2 per 100.000 persons each year. Especially an increase in the use of volar plating has been observed [9, 10, 12].

The goal of open reduction and plate fixation is to restore articular congruity and axial alignment to prevent post-traumatic osteoarthritis. Additionally, open reduction and plate fixation allows for early mobilisation and may theoretically lead to a more rapid recovery and better functional outcome [13, 14]. Especially in the young and working population, but also in the elderly patients, this could be an advantage. Moreover, redisplacement rates up to almost 60 % are encountered in patients treated with closed reduction and plaster immobilisation, especially in those with type C fractures [15–19]. However, with nonsurgical treatment the standard risks for undergoing a surgical procedure and the risk of hardware removal, tendon rupture and neurovascular damage are avoided. Moreover, we know that especially patients over 65 years of age have a lower disutility for painful malunion [20]. Though, plaster immobilisation is not without risks either. Pressure neuropathy of the superficial radial nerve, Complex Regional Pain Syndrome and stiffness of the wrist can occur.

In a recent randomised controlled trial, Bartl et al. compared ORIF with a volar locking plate with closed reduction and plaster immobilisation [21]. They included 149 patients of 65 years and older with a complete articular distal radius fracture (AO/OTA type C). Of all patients who were assigned to plaster treatment, 41 % was reassigned to secondary surgical fixation due to loss of reduction within 2 weeks after the initial treatment. The operative group had better range of motion and radiographic results after 3 months, however this was not accompanied by significantly better DASH and SF-36 scores. After 1 year no significant differences between both treatment arms were observed. Nevertheless, this study was terminated prematurely because the recruitment rate was much lower than projected. Moreover, Bartl et al. focussed on the elderly patients, so no conclusion can be drawn from this study for patients aged younger than 65.

To our knowledge, no randomised studies have been conducted to assess whether operative treatment with plate fixation is superior to non-operative treatment in patients with displaced complete articular distal radius fractures (AO/OTA type C) in patients from 18 to 65 years. In addition, the most up-to-date relevant Cochrane Review from 2003 states: “There is need for good quality evidence for the surgical management of these fractures” [22]. Therefore, with this randomised controlled trial we aim to determine the difference in functional outcome following open reduction and plate fixation compared with non-operative treatment with closed reduction and plaster immobilisation after one year of follow up.

## Methods/Design

### Study objectives

The primary objective is to evaluate the functional outcome of open reduction and internal plate fixation, compared with closed reduction and plaster immobilisation of displaced complete articular (AO/OTA type C) distal radius fractures in adults patients aged 18 to 65 years. The secondary objectives are to assess which treatment leads to less pain, a better range of motion and grip strength and less complications. Cost-effectiveness for both treatments is also evaluated.

### Study design

The VIPAR (Internal Plate Fixation versus Plaster in Complete Articular Distal Radius Fractures) trial is designed as a multicentre randomised controlled trial that will randomise between open reduction and internal plate fixation (intervention group) and closed reduction and plaster immobilisation (control group). Patients will be recruited in 15 Dutch hospitals.

### Study population

The study population will consist of all adult patients aged 18 to 65 years with a complete articular distal radius fracture (AO/OTA type C), diagnosed by an independent radiologist and classified based on radiography according to the AO/OTA classification of fractures. If radiography results do not provide sufficient information for an unambiguous classification, additional Computed Tomography (CT) of the wrist will be performed according to normal practice. Acceptable closed reduction has to be obtained immediately after presentation at the Emergency Department of one of the participating hospitals.

### Inclusion criteria

- Patients aged 18 to 65 years
- Complete articular (AO/OTA type C) displaced distal radius fracture
- Acceptable closed reduction on X-ray obtained immediately after admission to the Emergency Department (<12 h) defined, according to the Dutch National Guidelines [6], as:
  - Radial inclination ≥15°
  - Radial height (RH, also known as radial length) = distance between lateral most radial tip and ulnar surface: >5 mm, positive height shown
  - Volar angulation <20° or dorsal angulation <15°
  - Articular step-off or gap <2 mm. A step-off is defined as the loss of articular congruity of the distal radius perpendicular to the articular surface and parallel to the articular surface and a gap parallel to the articular surface [23]

### Exclusion criteria

- Open fracture of the distal radius
- Multiple trauma patients (Injury Severity Score (ISS) ≥16)
- Other fractures of the injured extremity (except for a fracture of the ulnar styloid process)
- Fracture of the contralateral wrist (distal radius, distal ulna or one of the carpal bones)
- Patients with impaired wrist function prior to injury due to arthrosis, rheumatoid arthritis, neurological disorders or malunion of the upper limb
- Patient suffering from disorders of bone metabolism other than osteoporosis (i.e. Paget’s disease, renal osteodystrophy, osteomalacia) or connective tissue disease or (joint) hyperflexibility disorders such as Marfan’s or Ehler Danlos
- Patients with insufficient understanding of the Dutch language to understand the study information and informed consent forms, the rehabilitation program and other treatment information as judged by the attending physician

### Interventions

After presentation to the Emergency Department, all patients will be treated with closed reduction and plaster immobilisation. Closed reduction will preferably be performed according to the Robert-Jones method [24] under local anaesthesia by means of a haematoma block with 20 cc Lidocaine 1 %. First continuous traction will be applied. Afterwards the deformity will be manually reduced and the wrist and hand will be immobilised in the reduced position. To verify the success of reduction, additional radiographs will be performed (see inclusion criteria). After adequate reduction has been confirmed, the wrist will be immobilised initially in a below-the-elbow single-cut plaster of paris, according to the Dutch guidelines [6]. After informed consent has been obtained, patients will be randomised either into the intervention group (ORIF) or into the control group (plaster immobilisation).

The control group will continue with plaster immobilisation. After approximately one week the single-cut plaster will be changed into a circular forearm plaster for five weeks immobilisation in total, according to the Dutch guidelines [6]. During plaster immobilisation advice and instructions on moving the uninvolved joints will be given. This includes flexion of the elbow and movement of the metacarpophalangeal (MCP) joints, the proximal interphalangeal (PIP) joints and the distal interphalangeal (DIP) joints. Additionally patients will be instructed to keep the arm high, preferably in a sling.

The intervention group will be treated with open reduction and locking plate fixation. All fractures will be fixed internally with a volar locking plate. This method employs a volar approach to the distal radius, according to Henry [25]. This approach implies an incision between the tendon of the flexor carpi radialis muscle and the radial artery. The advantages of the volar approach are the possibility of an uncomplicated extension of the distal part of the forearm and an optimal cover of the plate by soft tissue [26]. Furthermore, using this technique, the median nerve is not at risk. Additionally, fixation with a volar locking plate can be supported by a dorsal plate or radial column plate. This will be at discretion of the surgeon and depends on the fracture configuration and the position of the fragments. The same applies to the type and brand of the locking plate. Fracture reduction and screw placement will be fluoroscopically confirmed.

The surgery will be performed by a certified (orthopaedic) trauma surgeon. According to the current standard treatment protocol, antibiotic prophylaxis (Cefazoline, 1000 milligram intravenously) will be administered thirty minutes preoperatively [27]. Wound closure will be performed using standard techniques and will be at discretion of the surgeon. Wound inspection will be performed during the first follow-up visit at one week. Patients receive a pressure bandage for 24 h, after which they are instructed to use the affected extremity as pain allows. However, the first 6 weeks only non-weight bearing practice is allowed.

After treatment, for both the intervention and the control group, the same advice and instructions on moving the wrist after the treatment will be given, including instructions on practicing deviation, flexion and pronation and supination of the wrist. The choice for rehabilitation with help of a physiotherapist, will be at discretion of the patient.

### Outcomes

The primary outcome of this study is wrist pain and function expressed on the Patient-Rated Wrist Evaluation Score (PRWE) at one year follow-up. The PRWE is a validated questionnaire for assessing functional outcome and pain in patients with distal radius fractures [28]. In 1998 MacDermid et al. were the first to describe this score [29]. The PRWE consists of a 15-item questionnaire designed to measure two dimensions: wrist pain (*n* = 5 items) and function in activities of daily living (*n* = 10 items). Patients rate their levels of wrist pain and disability from 0 to 10 on two subscales: pain and function. The highest score on the subscale pain is 50, indicating worst pain, and the lowest score is zero, indicating no pain. The function score is the sum of the 10 items, divided by two. The total highest score is 100, indicating worst pain and severe impairment. The PRWE is the recommended questionnaire because it efficiently provides relevant data on pain and functional recovery in the affected wrist [30].

**Fig. 1:**
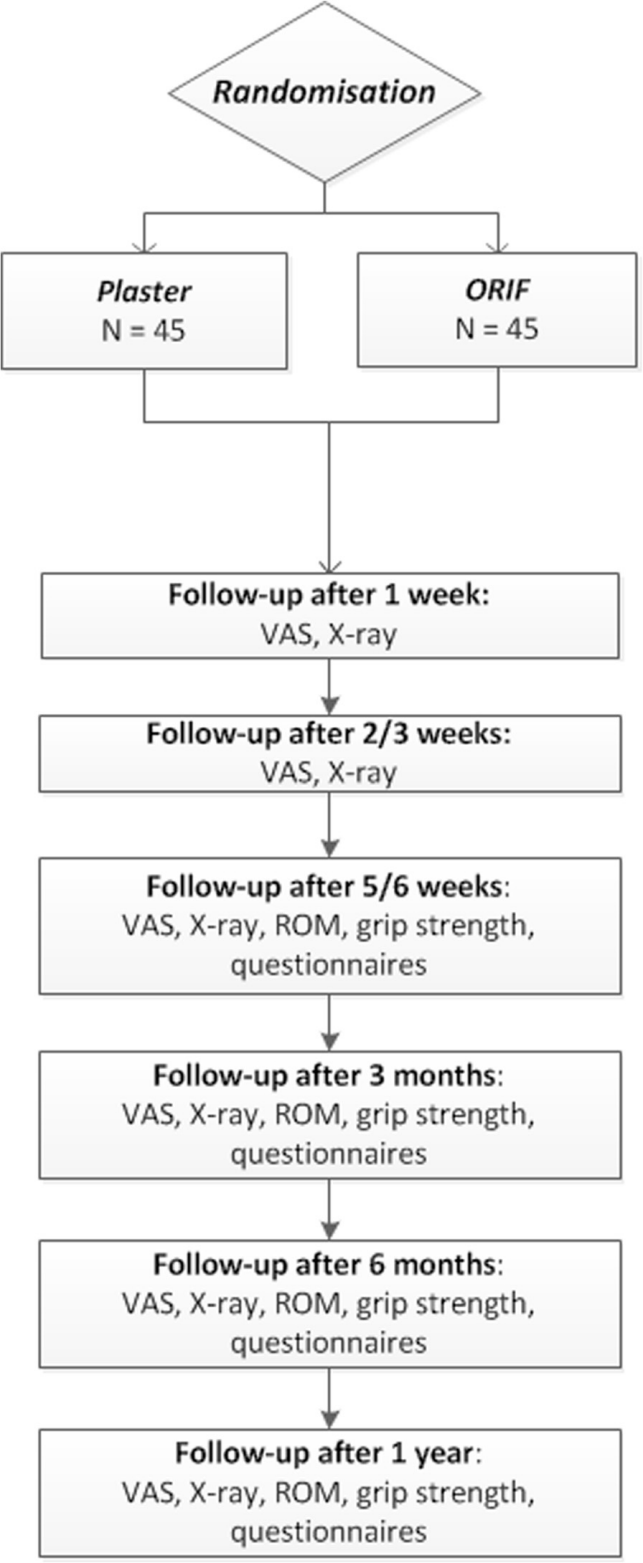
Follow-up visits
- Range of motion (ROM) of the wrist measured with a handheld goniometer of both the injured as the healthy wrist. ROM measurements will start in both groups at the third follow-up (at 5/6 weeks) (Fig. 1). In the control group, this will be the moment the plaster immobilisation ends. ROM measurements include pronation and supination, ulnar and radial deviation and palmar and dorsal flexion of the wrist.
- Grip strength, which will be measured on both sides with a Baseline dynamometer as the mean of three measurements. The difference in grip strength will be calculated as a percentage of the uninjured side. Grip strength measurements will start in both groups at the third follow-up (at 5/6 weeks) (Fig. 1).
- Radiographic parameters, such as radial inclination, radial length, volar or dorsal tilt and articular step-off and/or gap. This will be measured digitally in the Picture Archiving and Communication System (PACS) of the participating hospital on standard X-rays of the wrist. Radiographs will be obtained according to standardised procedures in three directions; posterior-anterior (PA), lateral carporadial and lateral. Radiographs will be performed at all follow-up visits.
- Number of crossovers. Crossovers include all patients who were randomised to treatment with plaster immobilisation (control group), but who require operative treatment due to loss of reduction during the first six weeks. Loss of reduction is defined as any displacement that no longer meets the previously mentioned inclusion criteria for acceptable reduction.
- The occurrence of complications, such as complaints in plaster, loss of reduction, superficial infection of the wound or deep infection of the plate, compartment syndrome, complex regional pain syndrome (CRPS) type 1 according to the Veldman [33] and the Budapest criteria [34, 35], tendinitis or rupture of the tendon, non-union, malunion, carpal tunnel syndrome and osteosynthesis material-related complications will be recorded.
- Cost-effectiveness and cost-utility measured with an economic evaluation questionnaire based on the EQ-6D and the Standard Form Health and Labour questionnaire. The aim of this economic analysis is to evaluate and compare the cost-effectiveness and cost-utility of closed reduction followed by plaster immobilisation and ORIF with a plate from a societal perspective. The SF-6D will be used to express the effect of quality of life in Quality Adjusted Life Years (QALY).

Secondary outcomes include:

- Wrist function, pain and disability as measured with the DASH-score. The DASH- score is measured in two components: the disability section (23 questions, scale 1-5) and the symptom section (7 questions, scale 1-5). The DASH questionnaire tests disability when performing a variety of activities in the past week because of arm, shoulder, or hand problems. Additionally, the DASH inquires about the severity of pain and tingling of the arm, shoulder or hand, as well as the effect of the upper limb injury on social activities, work, and sleep. The total score is calculated by a summation of the different scores on the two components, divided by the amount of questions, minus one. This amount is multiplied with 25. The highest total score is 100, indicating severe disability, and the lowest score is 0, indicating no disability. The DASH score is designed and validated to measure function and disability in patients with a distal radius fracture. [5, 30, 31].
- Quality of Life evaluated using the Short Form 36 (SF-36®) questionnaire. The SF-36 is a validated multipurpose, health questionnaire. The SF-36 consists of 36 questions representing eight different health domains [32]. These domains are divided into functional health and well-being scores, as well as psychometrically-based physical and mental health summary measures and a preference-based health utility index. From each domain, scores range from 0 to 100, with lower scores indicating poorer quality of life.
- Pain as indicated on a Visual Analogue Scale (VAS). On the VAS 0 implies no pain and 10 the worst possible pain. Patients will also be asked to give an estimation of the pain medication and the dosage taken during all follow-up visits (Fig. 1).

Since this analysis is from a societal perspective, direct health care costs, direct non- healthcare costs and indirect costs due to a distal radius fracture will be considered. Direct health care costs include treatment, follow up visits to medical specialist, any additional visits to general practitioner or other health care professionals, prescribed medication, professional home care and treatment of possible complications (Table 1). Direct non-healthcare costs include travel expenses to and from the hospital, over-the-counter medication, care provided by family or paid help and assistive devices. Indirect costs originating from loss of production or hours of inactivity due to immobilisation, pain or decreased function of the wrist will be included as well. These will be estimated using the Friction-Cost method that limits productivity loss to the friction period. This friction period is the period to recruit and train a replacement for the sick employee [36–38]. The Human Capital Approach, which is based on the total expected loss of production for an individual worker [38], will also be used to estimate costs. Compared with the Friction-Cost method, the Human Capital Approach will often overestimate costs from a societal perspective, especially in the long term [37, 39].

**Table 1:** Costs included in the economic evaluation

Direct health care costs	
Closed Reduction and Plaster immobilisation	
Open reduction and internal fixation	
Follow-up visits medical specialist	
Additional visits to health care professional	
Prescribed medication	
Professional home care	
Treatment and follow up of complications	
Physical therapy	
Direct non-health care costs	
Travel expenses to and from the hospital	
Over the counter medication	
Care provided by family or paid help	
Assistive devices	
Indirect costs	
Absenteeism from paid labour (per day)	
Absenteeism from unpaid labour	

Data regarding the use of health care resources will be assessed using four economic evaluation questionnaires (cost diaries) which patients will be asked to fill out during the trial at six weeks and three, six and twelve months [40]. The costs of direct health care related costs will be estimated by means of Dutch guidelines (DOT system).

### Randomisation

All patients diagnosed with a displaced complete articular AO/OTA type C distal radius fracture will be invited to participate in this study. Patients will be approached by the investigators to participate in this study after adequate reduction of the fracture has been obtained. Patients will receive a patient information sheet with both the contact information of the coordinating investigator and the independent expert. Possible participants will have till the first outpatient appointment to decide to participate or not. This first outpatient appointment will be about 7 days after the initial trauma. If a patient decides to participate, oral and written informed consent will be obtained. Possible participants can be approached and included in this study up to the first outpatient visit.

After obtaining informed consent, patients will be randomised into either the intervention group (open reduction and plate fixation) or the control group (closed reduction and plaster immobilisation). In order to avoid any potential imbalances between the two treatment groups, patients will be randomised in two strata according to age: 18 to 40 and 41 to 65 years.

To ensure concealment of allocation, randomisation will be performed by means of a mixed block, computerised randomisation. The distribution and order of the block sizes are unknown to the researchers, who therefore remain blinded to the allocation of the next subject throughout the whole study. Surgery will be performed within two weeks after randomisation. All the participating centres can perform the randomisation on the study website (www.viparstudie.nl), where each centre has his own personal username and password. After randomisation the allocation is shown on the website.

### Blinding

Randomisation status will not be blinded since the assigned treatment involves a surgical procedure.

### Sample size calculation and data analysis

The sample size calculation is based on our primary endpoint, the PRWE score. The mean PRWE score after open reduction and volar locking plate fixation after one year of follow-up in patients aged over 18 years with a dorsally displaced distal radius fracture is 15 with a standard deviation of 17 [41]. This figure was measured in a patient population in which 33 % suffered from a displaced complete articular distal radius fracture (AO/OTA type C fracture). The same mean and standard deviation were found in a study of MacDermid et al. [42].

The minimal clinical important difference (MCID) is the minimal change in score that is considered as meaningful to patients [30]. The MCID of the PRWE score is 14 points according to Sorensen et al. [43]. Thus, to demonstrate a difference that is clinically relevant, we need to power on a difference of 14 points. Any difference smaller than this is not clinically relevant.

Therefore at α = 0.05 % and a power of 90 %, we would require 64 patients in total and 32 per treatment arm. For safety measures, to correct for deaths and with an expected lost to follow-up of 10 %, 90 patients (45 patients in each arm) will be included. From a separate study being conducted by our research group at two teaching hospitals, it was found that of the 494 distal radius fractures encountered in one year, 126 (25 %) were an AO/OTA type C [2]. Therefore we estimate to require a maximum of 2 years to include and follow-up the patients in this trial.

All patients will be analysed according to the results of the randomisation (intention-to-treat). Patient characteristics at baseline, such as gender and age, will be described by general descriptive statistics. Differences in the primary outcome (PRWE at one year) between the two groups will be analysed using the Unpaired *T*-test (if normally distributed) or the Mann–Whitney *U* test (if not normally distributed). Trends in PRWE-scores among the different time points will be assessed using multiple linear regression. The secondary outcomes; DASH-scores, quality of life (SF-36), pain (as indicated on a Visual Analogue Scale (VAS)), range of motion (ROM) and grip strength will be analysed in a similar manner. Differences between the two treatment groups in radiological outcomes, crossovers and complication rates will be analysed using the Chi-square test or the Fischer’s Exact test (in case the expected incidence was less than five). Subgroup analyses will be performed on gender and age (18 to 40 years and 41 to 65 years) for all outcomes.

### Ethics

This study will be conducted according to the principles of the Declaration of Helsinki version 64, October 2013 and in accordance with the Medical Research Involving Human Subjects Act (WMO) and ‘Good Clinical Practice’ guidelines.

Data will be stored in two separate files. One data set will contain coded patient information and a second set medical history linked to these codes. The key to the code will be safeguarded by the coordinating investigator. Data will be stored and kept for fifteen years according standard guidelines.

The Medical Ethical Review Committee of the Academic Medical Center in Amsterdam has approved the protocol on January 20, 2015. The board of directors of 14 Dutch participating centres approved for local feasibility. The participating centres are listed in appendix 1.

Prior to start, the trial has been registered at the Netherlands Trial Register (NTR4915).

## Discussion

Since the treatment allocation involves a surgical procedure, randomisation status will not be blinded. Our research group discussed the idea to let the researcher, who is assessing the outcomes at one year, be unaware of the treatment allocation. However this is practically not possible since all follow-up visits are realised by one or two researchers. At final follow-up, these researchers will have assessed the participants multiple times in the past year and will therefore be aware of their treatment allocation. Although this arrangement makes blinding impossible, the continuity of the observer is one of the strengths of this study and this will minimise intra-observer variation in the outcome measurements.

Additionally, for the measurement of the radiographic parameters we used the PACS of the participating hospitals. Although PACS is good to measure angles (inclination and dorsal or volar angulation) and the inter- and intra-observer reliability are substantial and moderate respectively [44], the linear measurement (radial length and gap or step-off) in PACS is less exact.

Last, due to the fact that patients will be analysed according to the intention-to-treat principle, possible crossovers will be analysed in the group they were allocated to.

## Conclusion

Although displaced intra-articular distal radius fractures are common, there is still no evidence on the optimal treatment for these fractures in patients aged 18 to 65 years. With this randomised controlled trial we aim to determine the difference in functional outcome following open reduction and plate fixation compared with non-operative treatment with closed reduction and plaster immobilisation.

## Appendix 1: List of participating centres

Academic Medical Center, Amsterdam

Maasstad Hospital, Rotterdam

Westfriesgasthuis, Hoorn

Radboud University Medical Center, Nijmegen

Diakonessenhuis, Utrecht

Groene Hart Hospital, Gouda

Maxima Medical Center, Veldhoven/Eindhoven

BovenIJ Hospital, Amsterdam

Reinier de Graaf Gasthuis, Delft

Hospital Amstelland, Amstelveen

Catharina Hospital, Eindhoven

Onze Lieve Vrouwe Gasthuis, Amsterdam

Alrijne Hospital, Leiderdorp

Flevoziekenhuis, Almere

Hospital Rivierenland, Tiel

## Abbreviations

AMC: Academic Medical Center Amsterdam
AO/OTA: Arbeitsgemeinshaft für Osteosynthesefragen/Orthopedic Trauma Association
CT: computed tomography
DASH: disability of the arm, shoulder and hand
GRC: global rating of change
ISS: injury severity score
MCID: minimal clinical important difference
ORIF: open reduction and internal fixation
PA: posterior anterior
PACS: picture archiving and communication system
PRWE: patient rated wrist evaluation
QoL: quality of life
RH: radial height or radial length
ROM: range of motion
SD: standard deviation
SF-36: short form 36
VAS: visual analogue scale
